# A Modified Dura Puncture Procedure to Reduce Brain Shift in Deep Brain Stimulation Surgery: One Institution's Experience

**DOI:** 10.3389/fneur.2022.845926

**Published:** 2022-02-28

**Authors:** Yu-Xi Wu, Wei Xiang, Jia-Jing Wang, Xiao-Ming Liu, Dong-Ye Yi, Han Tian, Hong-Yang Zhao, Xiao-Bing Jiang, Peng Fu

**Affiliations:** ^1^Department of Neurosurgery, Union Hospital, Tongji Medical College, Huazhong University of Science and Technology, Wuhan, China; ^2^Department of Radiology, Union Hospital, Tongji Medical College, Huazhong University of Science and Technology, Wuhan, China

**Keywords:** dural puncture procedure, brain shift, electrode implantation accuracy, deep brain stimulation, modified procedure

## Abstract

**Objective:**

The therapeutic effect of deep brain stimulation (DBS) surgery mainly depends on the accuracy of electrode placement and the reduction in brain shift. Among the standard procedures, cerebrospinal fluid (CSF) loss or pneumocephalus caused by dura incision (DI) is thought to be the main reason for brain shift and inaccuracy of electrode placement. In the current study, we described a modified dura puncture (DP) procedure to reduce brain shift and compare it with the general procedure of DBS surgery in terms of electrode placement accuracy.

**Materials and Methods:**

We retrospectively analyzed a series of 132 patients who underwent DBS surgery in Wuhan Union Hospital from December 2015 to April 2021. According to the different surgery procedures, patients were classified into two cohorts: the DI group (DI cohort) had 49 patients who receive the general procedure, and the DP group (DP cohort) had 83 patients who receive the modified procedure. Postoperative pneumocephalus volume (PPV) and CSF loss volume, electrode fusion error (EFE), and trajectory number were calculated. Meanwhile, intraoperative electrophysiological signal length (IESL), electrode implantation duration, and other parameters were analyzed.

**Results:**

In the current study, we introduced an improved electrode implantation procedure for DBS surgery named the DP procedure. Compared with the general DI cohort (*n* = 49), the modified DP cohort (*n* = 83) had a shorter electrode implantation duration (*p* < 0.0001), smaller PPV, lower CSF leakage volume (*p* < 0.0001), and smaller EFE (*p* < 0.0001). There was no significant difference in IESL (*p* > 0.05) or adverse events (perioperative cerebral haematoma, skin erosion, epilepsy, *p* > 0.05) between the two cohorts.

**Conclusion:**

The DP procedure is a modified procedure that can reduce brain shift and ensure implantation accuracy during DBS surgery without adverse events.

## Introduction

Deep brain stimulation (DBS) was first established to improve the motor symptoms of Parkinson's disease (PD) and has been gradually approved to treat an increasing number of other disorders (1, 2). Accurate electrode implantation into the target nucleus is a key determinant of clinical benefits (3). To confirm the millimetric accuracy, intraoperative microelectrode recording (MER) is used to define the borders of the target nucleus, test stimulation is routinely performed to check for side effects and assess the surgical effects, and intraoperative MRI provides high-resolution visualization of the neuroanatomy (4, 5). In addition to these confirmative measurements, many surgical details, such as surgical skills, team cooperation, patient position, surgical duration, burr hole localization, dura incision (DI) size, and temporary burr hole closure, were established as the general procedure to ensure accurate implantation in several experienced DBS centers (6–8).

As a general surgical procedure, a DI is a necessary step to place electrodes under direct vision (3). Generally, surgeons perform the “DI” procedure: cross-cut the dura, expose the cortex, and implant the catheter. The DI inevitably results in cerebrospinal fluid (CSF) loss and pneumocephalus, and this causes brain movement and deformation in terms of its anatomical and physiological position, such as inaccurate electrode placement and nonideal clinical outcomes of DBS surgery (4, 9). This situation worsens with increasing amounts of CSF loss and air inflow, such as second electrode implantation or a long duration of surgery after the DI (10).

To reduce the brain shift caused by the DI, we gradually modified the general procedure of DBS surgery in Wuhan Union Hospital and developed a “dura puncture, DP” from October 2017 to March 2018. Herein, we report the data of 49 patients who underwent DBS surgery following the general procedure from December 2015 to October 2017 and 83 patients who underwent DBS surgery following the modified procedure from March 2018 to April 2021. The brain shift and clinical characteristics were investigated in a retrospective study.

## Materials and Methods

### Patient Population

The current study retrospectively analyzed a series of one hundred thirty-two patients who underwent DBS surgery in Wuhan Union Hospital from December 2015 to April 2021. The 49 patients who underwent DBS surgery following the general procedure from December 2015 to October 2017 were included in the DI group (DI cohort), and the 83 patients who underwent DBS surgery following the modified procedure from March 2018 to April 2021 were included in the DP group (DP cohort). This study was approved by the local ethics committee of Tongji Medical College, Huazhong University of Science and Technology. All patients were provided informed consent for study inclusion before the DBS surgery.

### Surgical Procedures

All 132 surgeries were performed by one neurosurgical team (PF, WX, HYZ, XBJ, and JR). From October 2017 to March 2018, 16 patients underwent DBS surgery in our department, and the surgical procedure was modified gradually during this period, so they were excluded from the study.

For the DI cohort, the surgical procedure was as described previously, and T1- and T2-phase weighted brain MRI was obtained for the surgical plan to determine the target and paths before surgery by using the SurgiPlan Workstation (Elekta Instruments AB, Stockholm, Sweden). Gadolinium-containing contrast agents were used in patients who underwent MRI or magnetic resonance angiography (MRA) scans. Subsequently, CT scanning was performed after a Leksell head stereotactic frame was mounted under local anesthesia. The electrode implantation coordinates were determined by merging the initial target parameters on MRI and the frame coordinates on CT. The patients were kept in the supine position, and surgery was performed as follows: skin incision, drill burr hole, cross-cut the dura, expose the cortex, and implant the catheter under direct vision. With the guidance of electrophysiological recording, quadripolar DBS electrodes were bilaterally implanted into the initial targets, and then the implantable pulse generator (IPG) was connected to the electrodes.

For the DP cohort, some steps were added or modified. Additional enhanced T1-weighted brain MRI was performed using the same scanning parameters to build an image of the intracranial vessels, and then the planned puncture paths were kept away from the intracranial vessels to decrease the bleeding. After making the burr holes, dura and arachnoid membrane puncture was performed by adequate electrocoagulation of the puncture catheter with a monopole at 20 J. Water flushing was used to remove any blood, and then gel foam and fibrin glue were filled into the burr holes to block CSF loss and air inflow.

### Data Acquisition

Data were collected and evaluated by two independent neurosurgeons (YXW and HT) who were involved in the surgery. Before the surgery, the clinical characteristics of all of the patients were recorded, and MRI/CT scans were taken routinely. During the surgery, relevant parameters, such as intraoperative electrophysiological signal length (IESL), stimulating electrode placement depth, the timing of the electrode adjustment, and implantation duration, were recorded. After the surgery, a postoperative 2 mm cranial CT scan was obtained within 8 h. Then, the postoperative pneumocephalus volume (PPV) and CSF loss volume were determined by volumetric segmentation using Osirix v.3.6.1 software. The PPV was calculated on air-present CT using the formula: volume = (A × B × C)/2, where A is the slice thickness of cranial air, B is the largest diameter of the air volume on a single slice, and C is the largest diameter of air measured orthogonally from the cortex to the skull. The magnitude of cortical brain shift (CBS), which equals C, was also examined.

At the same time, postoperative cranial CT and preoperative MR were fused to calculate the electrode fusion error (EFE), which is the maximum distance from the implanted electrode to the planned path, on the SurgiPlan Workstation. Before initiating the IPG programming, a 2 mm cranial CT was scanned again and imported to Lead-DBS software (www.lead-dbs.org) and reconstructed to show the three-dimensional electrode locations with preoperative MRI.

The following clinical characteristics were also collected for further analysis: age, sex, hospitalization time, DBS target, electrode implantation duration, perioperative cerebral haematoma, skin erosion, and epilepsy.

### Statistical Analysis

Metrology data were statistically analyzed to obtain mean and median values. Student's *t*-test was used to compare the means of continuous variables between two groups. Target precision and bias were compared between the two groups using the chi-square test. Probability values <0.05 were defined as statistically significant. All procedures were performed with GraphPad Prism 8.

## Results

### Patient Characteristics

The current study retrospectively analyzed one hundred thirty-two consecutive patients (60 women and 72 men) who underwent DBS surgery in Wuhan Union Hospital from December 2015 to February 2021. All 132 patients completed at least 3 months of follow-up. From October 2017 to March 2018, the surgical procedure was modified gradually with continuous improvement and innovation of the surgical experience, and 16 patients who underwent DBS surgery in our department were excluded from this study because they did not meet the inclusion criteria completely.

According to the surgical procedures and timetable, these 132 patients were classified into two cohorts (DI cohort and DP cohort). No significant difference in age, sex, or hospitalization time was observed between these two groups (*p* > 0.05). In the DI cohort, 39 patients with PD underwent subthalamic nucleus (STN)-DBS surgery under local anesthesia and 10 patients with dystonia underwent globus pallidus internus (Gpi)-DBS surgery under general anesthesia. In the DP cohort of 61 patients with PD and 22 patients with dystonia, 72 patients underwent STN-DBS surgery, 11 patients underwent Gpi-DBS surgery, 13 patients underwent local anesthesia surgery, and 70 patients underwent general anesthesia surgery. The clinical characteristics of all 132 patients in the two cohorts who underwent DBS surgery in Wuhan Union Hospital are shown in Table 1.

**Table 1:** Clinical characteristics of 132 patients who underwent DBS in the Wuhan Union Hospital.

**Characteristics**		**DI cohort**	**DP cohort**	**Total**	***P*-value**
Number		49	83	132	
Age (Years)		48.36 ± 21.23	53.64 ± 20.30	51.36 ± 26.71	0.4214
Sex					0.9214
	Male	27	45	72	
	Female	22	38	60	
Hospitalization time (Days)		11.8 ± 3	10.8 ± 2.2	11.5 ± 2.6	0.2970
Primary diagnosis
	PD	39	61	100	
	Dystonia	10	22	32	
Anesthesia method
	Local anesthesia	39	13	52	
	General anesthesia	10	70	80	
Electrode target
	STN	39	72	111	
	Gpi	10	11	21	
Operative adverse events
	Perioperative cerebral hematoma	1 side in 98 sides	1 side in 166 sides		
	Skin erosion	0	0		
	Epilepsy	0	0		
Stimulation side effects		0	0		

*DBS, Deep Brain Stimulation; STN, subthalamic nucleus; Gpi, globus pallidus interior; PD, Parkinson's Diseases*.

### Surgical Technique Notes About the Modified DP Procedure in DBS Surgery

**Figure 1:**
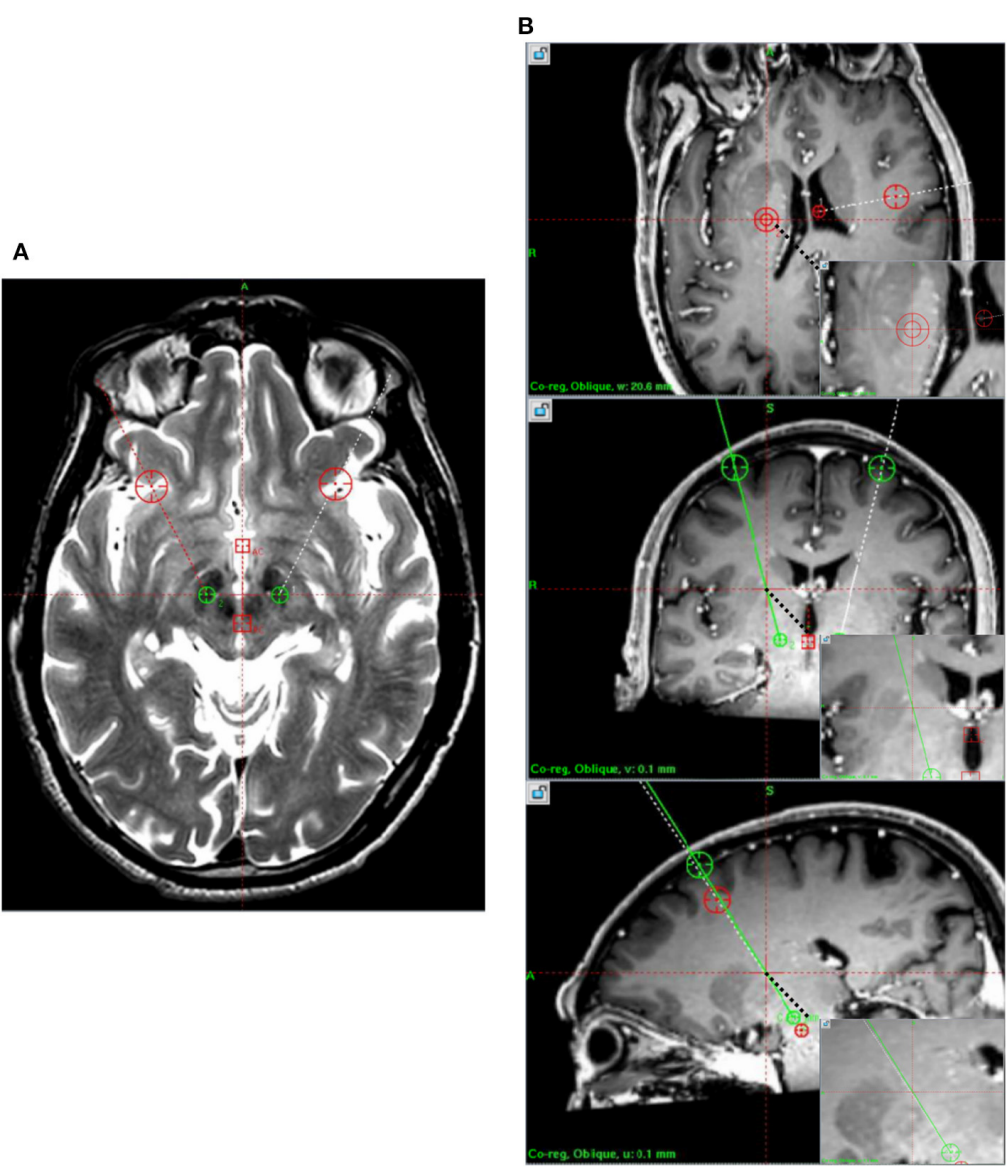
Preoperative magnetic resonance imaging. **(A)** T2 images demonstrate the target. **(B)** T1 enhanced the discrimination of the blood vessels.

In the general procedure of DBS surgery, dural cross-incision could provide direct vision to expose the cortex and implant the puncture catheter, but CSF loss and air inflow could occur. In the modified procedure, we opened the dura and arachnoid membrane by adequate electrocoagulation with a monopole at 20 J. The puncture catheter could deliver energy from the monopole and penetrate through the dura and arachnoid membrane, as shown in Supplementary Video 1. Water flushing could rapidly remove any blood. Then, gel foam and fibrin glue are filled into the burr holes to block CSF loss and air inflow, as shown in Figure 1A. This modified step could prevent CSF loss and brain shift, so the electrodes could be implanted along the paths in accordance with the plan.

To decrease the rate of DP-induced hemorrhage, a similar method of stereotactic electroencephalography surgery was performed in the modified procedure of DBS surgery. Additional enhanced T1-weighted brain MRI with the same parameters was obtained to build an image of the intracranial vessels, and then the electrode trajectory was kept away from the intracranial vessels in the surgery plan, as shown in Figure 1B. This additional step could avoid perioperative cranial hemorrhage without direct vision to the maximum extent.

### Modified DP Procedure Reduces Brain Shift in DBS Surgery

The selected cases present a comparison of brain shifts between the DI cohort and the DP cohort, as shown in Table 2. In the implantation period, the one-trajectory rate of MER was similar on the first side between these two cohorts (*p* = 0.1279), and it was lower in the DP cohort on the second side (*p* = 0.0018). Compared with the DP cohort, the DI cohort always took a long time to obtain satisfactory intraoperative MER signs or test stimulation effects (2.02 ± 0.77 h vs. 1.27 ± 0.56 h, *p* < 0.0001). The IESLs of the STN (5.16 ± 1.12 mm vs. 5.42 ± 0.82 mm) and Gpi (6.03 ± 2.76 mm vs. 6.86 ± 1.95 mm), which were used to identify the implanted location of electrodes, were not different between the two cohorts (*p* > 0.05).

**Table 2:** Brain shift-related parameters between two cohorts of 132 patients.

**Characteristics**		**DI cohort**	**DP cohort**	***P*-value**
**One-trajectory rate of MER**				
	1^st^ side	47/49	82/83	0.2840
	2^nd^ side	39/49	80/83	0.0018
Electrode implantation duration (Bilateral average, hours)		2.02 ± 0.77	1.27 ± 0.56	*p* < 0.0001
**Intraoperative electrophysiological signal length (Bilateral average, mm)**				
	STN	5.16 ± 1.12	5.42 ± 0.82	0.1279
	Gpi	6.03 ± 2.76	6.86 ± 1.95	0.0556
Postoperative pneumocephalus volume (Bilateral average, ml)		12.5 ± 11.3	3.1 ± 3.7	*p* < 0.0001
Cortical brain shift (Bilateral average, mm)		4.5 ± 3.9	0.4 ± 1.9	*p* < 0.0001
Electrode fusion error (Bilateral average, mm)		0.86 ± 0.34	0.36 ± 0.36	*p* < 0.0001

*STN, subthalamic nucleus; Gpi, globus pallidus interior*.

**Figure 2:**
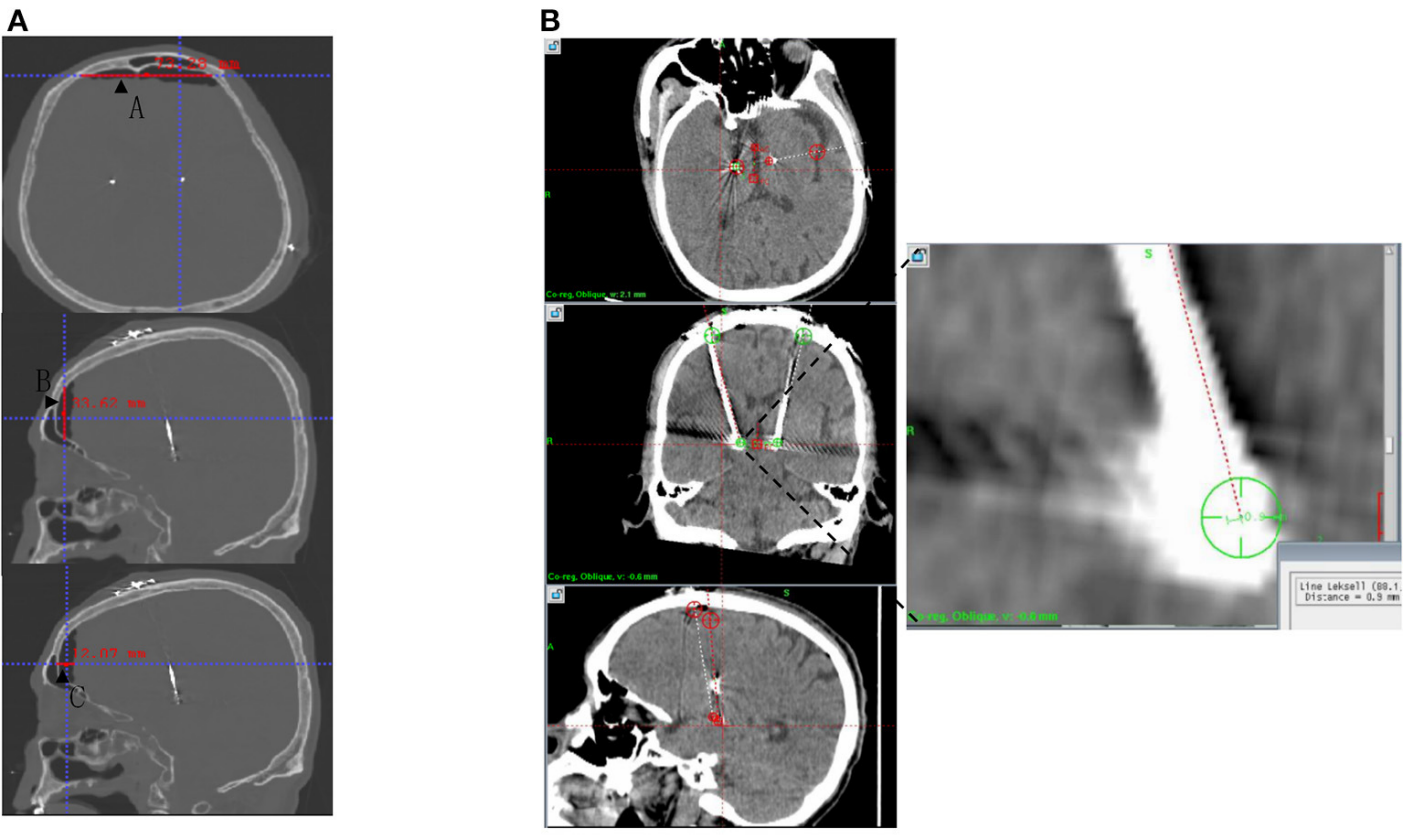
Postoperative CT data measurement. **(A)** PPV and CBS were calculated from A, B, and C under the same coordinate system measured after postoperative CT postprocessing. **(B)** Postoperative CT was fused with preoperative planning to measure EFE. CBS, Cortical brain shift; CT, computed tomography; EFE, Electrode fusion error; PPV, Postoperative pneumocephalus volume.

**Figure 3:**
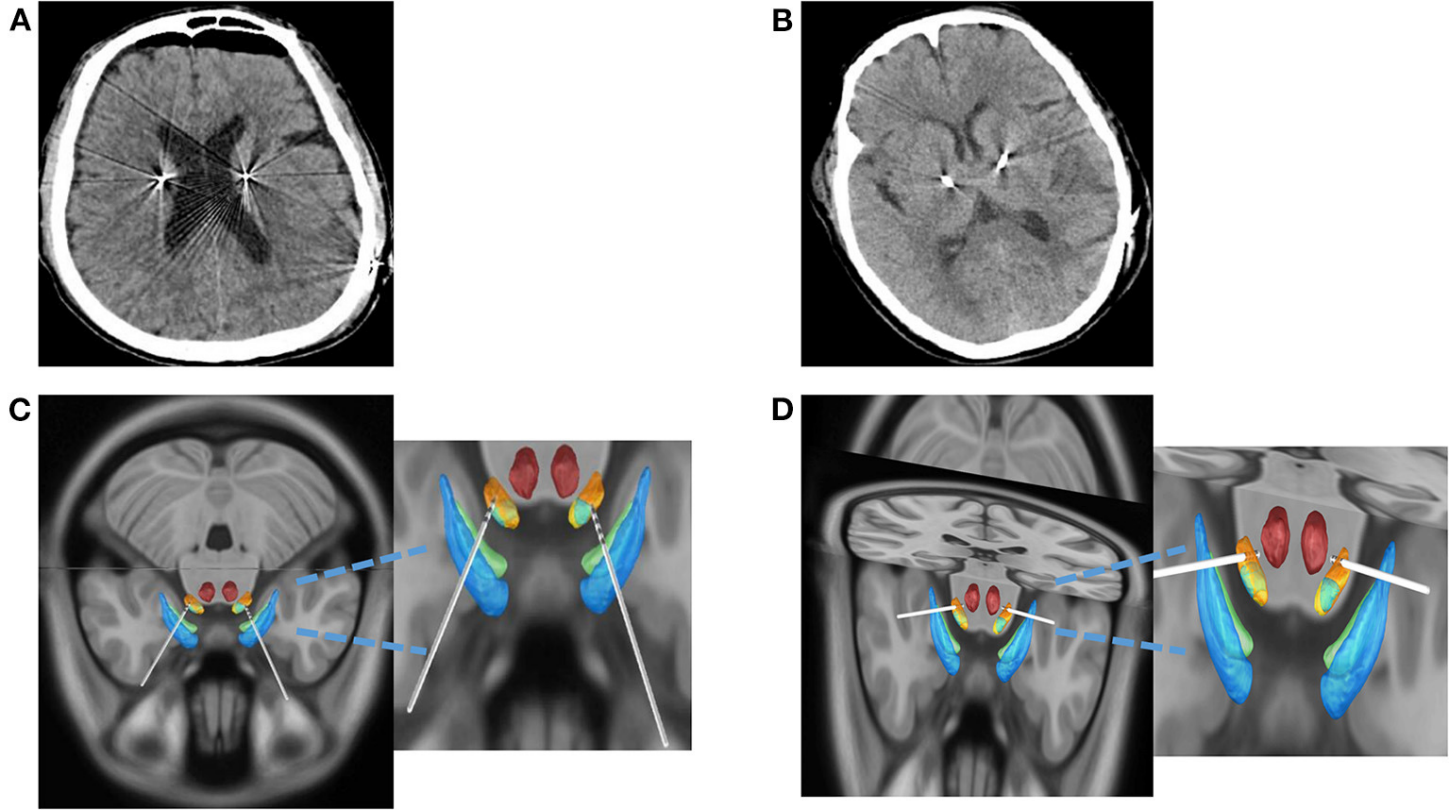
CSF loss and offset of electrode implantation were compared between the two groups. **(A)** Postoperative CT in DI Group. **(B)** Postoperative CT in DP Group. **(C)** The images were reconstructed by the DI group using Lead-DBS software. **(D)** The images were reconstructed by the DP group using Lead-DBS software. CT, computed tomography; CSF, cerebrospinal fluid; DI, dura incision; DP, dura puncture.

A postoperative cranial CT was always taken within 8 h to check for the possibility of intracranial hemorrhage and pneumatosis. The PPV and CSF loss volume were much lower in the DP cohort (3.1 ± 3.7 ml) than in the DI cohort (12.5 ± 11.3 ml, *p* < 0.0001). The CBS was also lower in the DP cohort (4.5 ± 3.9 mm vs. 0.4 ± 1.9 mm, *p* < 0.0001), even though some cases showed no significant postoperative intracranial pneumatosis in the DP cohort. When the postoperative CT IS fused with perioperative MR to calculate the implantation error, as shown in Figure 2, the DP cohort implanted electrodes with a shorter EFE (0.86 ± 0.34 mm vs. 0.36 ± 0.36 mm, *p* < 0.0001). In brief, the modified procedure could reduce the brain shift in DBS surgery, as shown in Figure 3.

### Safety of the Modified DP Procedure in DBS Surgery

Intracranial hemorrhage is a concern in DBS surgery, especially when a puncture catheter is implanted without direct vision in the modified procedure (4). Electrode implantation-related hemorrhage occurs on only one side in a total of 166 sides in the DP cohort, while there is also one affected side in a total of 98 sides in the DI cohort, as shown in Table 1. These two patients did not require any further surgical intervention. All 132 patients finished the initial IPG programming, and at least 3 months of follow-up, there was no skin erosion or stimulation side effects (such as worsening of balance, motor function, and dysarthria in either cohort. Furthermore, arachnoid membrane puncture and cortex damage by electrocoagulation of the puncture catheter did not seem to increase the incidence of epilepsy in the DP cohort.

## Discussion

Accurate electrode implantation into the target nucleus determines the clinical benefits of DBS surgery, and brain shifts caused by CSF loss and air inflow pose a real challenge (11). In this study, we introduced a modified DP procedure for DBS surgery that could avoid CSF loss postoperatively and limit pneumatosis; moreover, the modified procedure did not increase the operative adverse events or stimulation side effects.

In general, brain shifts produced by CSF loss and iatrogenic pneumocephalus could affect the efficacy of DBS surgery (1, 12). A systematic review declared a clear link between pneumocephalus volume reduction and target accuracy (13, 14). Kim et al. found that CSF loss and postoperative pneumocephalus might result in electrode dislocation due to brain expansion and air reabsorption and then alter the long-term efficacy of electrical stimulation (15). Okun et al. reported that postoperative pneumocephalus could induce a 46% treatment failure (16). Several methods have been applied to reduce the adverse effects of CSF loss and brain shift, but the results are complex. Reducing the drilling diameter failed to truly improve the pneumocephalus and reducing the operation time did not have any direct effect on DBS electrode target accuracy (6–8, 17, 18). A direct durotomy with the trajectory of multiple microelectrodes provides a more precise picture of the deep neuronal architecture (19). Consistent with the report of Massimo et al., our center developed a modified DP procedure for DBS surgery from October 2017 to March 2018.

In the modified procedure, the detection and avoidance of blood vessels in the preoperative plan and modified DP improvements were the two key points. Haemorrhagic complications were the most important concerns when we modified the procedure (20). Similar to stereo-encephalography (SEEG), the modified procedure also places electrodes without direct vision. Gadolinium-enhanced T1 images are used to identify vessels that should be avoided during DBS path planning. Electrode conflicts with vessels 1 mm in size could be detectable and thus could potentially be excluded from consideration during DBS planning (20, 21). Meanwhile, we always flushed the subdural space with water and checked the vessels after the puncture catheter penetrated the cortex. Elias et al. reported a haemorrhagic complication rate of 0.7% in DBS surgery. A similar haemorrhagic complication rate was achieved in our center, and the modified procedure did not increase the hemorrhage rate in the DP cohort (11). Several surgical improvements we made could effectively decrease the CSF loss and air inflow (8, 18). First, the dura was opened by monopolar electrocoagulation in the modified procedure instead of cross-cutting as in the general procedure. The dura hole size was much smaller in the case of monopolar electrocoagulation than that of cross-cutting. Second, monopolar electrocoagulation could also open the arachnoid membrane and coagulate the arachnoid membrane to the cortex, and the cavum subarachnoid was closed again. Third, gel foam and fibrin glue were filled into the burr holes after monopolar electrocoagulation, which could prevent CSF loss and air inflow (2, 6).

The current study reported brain shift-related parameters of 83 patients who underwent DBS surgery in our center following the modified procedure from March 2018 to April 2021. Compared with 49 patients following the general procedure from December 2015 to October 2017, the DP cohort had a lower one-trajectory rate of MER on second side electrode implantation, a shorter surgical duration, a smaller PPV and CSF loss volume, a shorter CBS, and a lower EFE. At the same time, the DP cohort did not show increased operative or stimulation-related adverse effects. In brief, the modified procedure in our center is a beneficial improvement for DBS surgery to avoid CSF loss and reduce brain shift without increased operative complications. Recently, DBS under general anesthesia and DBS with different stimulative targets for multiple disorders have been performed in an increasing number of experienced neurosurgical centers, and CSF loss and brain shift have become topics of concern in DBS surgery (22). The modified procedure provides the chance of DBS under general anesthesia and DBS with different stimulative targets to complete electrode implantation as a surgical plan.

The limitations of this study should be mentioned and improved in the future. First, this is a retrospective single-center study with a small sample size, and more DBS centers with more cases or randomized controlled trials are still needed to validate our technique. Second, patients with different treatment indications, different stimulative targets, and anesthesia methods were included in this study. The relationships between indications, targets, brain shift, and clinical outcomes were not analyzed (21, 23). Third, the brain shift-related parameters in this study or other studies are not well established to analyse the accuracy of electrode placement, and a more precise surveying system should be developed to evaluate CSF loss and brain shift.

## Conclusions

In conclusion, the current study described a modified dura puncture procedure of DBS surgery with a reduction in brain shift and tolerable adverse events, thus ensuring accurate electrode implantation and predictable clinical benefits. This technique is promising for DBS under general anesthesia and DBS with different stimulative targets to treat multiple disorders.

## Data Availability Statement

The raw data supporting the conclusions of this article will be made available by the authors, without undue reservation.

## Ethics Statement

The studies involving human participants were reviewed and approved by the local Ethics Committee of Tongji Medical College, Huazhong University of Science and Technology. Written informed consent to participate in this study was provided by the patient/participants or patient/participants legal guardian/next of kin.

## Author Contributions

PF and WX concepted and designed the study. Y-XW, X-ML, J-JW, HT, and D-YY contributed to acquisition of data. Y-XW, WX, and J-JW analyzed and interpreted the data. Y-XW and PF drafted the article. H-YZ, X-BJ, WX, and PF revised the article. PF reviewed submitted version of the manuscript. All authors contributed to the article and approved the submitted version.

## Conflict of Interest

The authors declare that the research was conducted in the absence of any commercial or financial relationships that could be construed as a potential conflict of interest.

## Publisher's Note

All claims expressed in this article are solely those of the authors and do not necessarily represent those of their affiliated organizations, or those of the publisher, the editors and the reviewers. Any product that may be evaluated in this article, or claim that may be made by its manufacturer, is not guaranteed or endorsed by the publisher.

## Supplementary Material

Supplementary Video 1 The modified dura puncture procedure included monopolar electrocoagulation, water flushing, gel foam, and fibrin glue plugging.
